# Image calibration and analysis toolbox – a free software suite for objectively measuring reflectance, colour and pattern

**DOI:** 10.1111/2041-210X.12439

**Published:** 2015-08-06

**Authors:** Jolyon Troscianko, Martin Stevens

**Affiliations:** ^1^ Centre for Ecology & ConservationCollege of Life & Environmental SciencesUniversity of ExeterPenryn CampusPenrynTR10 9FEUK

**Keywords:** animal coloration, camera calibration, colour measurement, colour vision, communication, cone‐catch quanta, image processing, pattern analysis, signalling, spectrometer

## Abstract

1. Quantitative measurements of colour, pattern and morphology are vital to a growing range of disciplines. Digital cameras are readily available and already widely used for making these measurements, having numerous advantages over other techniques, such as spectrometry. However, off‐the‐shelf consumer cameras are designed to produce images for human viewing, meaning that their uncalibrated photographs cannot be used for making reliable, quantitative measurements. Many studies still fail to appreciate this, and of those scientists who are aware of such issues, many are hindered by a lack of usable tools for making objective measurements from photographs.
2. We have developed an image processing toolbox that generates images that are linear with respect to radiance from the RAW files of numerous camera brands and can combine image channels from multispectral cameras, including additional ultraviolet photographs. Images are then normalised using one or more grey standards to control for lighting conditions. This enables objective measures of reflectance and colour using a wide range of consumer cameras. Furthermore, if the camera's spectral sensitivities are known, the software can convert images to correspond to the visual system (cone‐catch values) of a wide range of animals, enabling human and non‐human visual systems to be modelled. The toolbox also provides image analysis tools that can extract luminance (lightness), colour and pattern information. Furthermore, all processing is performed on 32‐bit floating point images rather than commonly used 8‐bit images. This increases precision and reduces the likelihood of data loss through rounding error or saturation of pixels, while also facilitating the measurement of objects with shiny or fluorescent properties.
3. All cameras tested using this software were found to demonstrate a linear response within each image and across a range of exposure times. Cone‐catch mapping functions were highly robust, converting images to several animal visual systems and yielding data that agreed closely with spectrometer‐based estimates.
4. Our imaging toolbox is freely available as an addition to the open source ImageJ software. We believe that it will considerably enhance the appropriate use of digital cameras across multiple areas of biology, in particular researchers aiming to quantify animal and plant visual signals.

## Introduction

Objective measures of appearance provide vital information in numerous areas of biology. This ranges from studies investigating the mechanisms and functions of visual signals, communication and camouflage across many taxonomic groups, work investigating human and non‐human vision, through to palaeontology and more applied areas, such as forensics and medical diagnoses. The ability to analyse such traits rigorously is essential if we are to understand their evolution and function. There exists a range of methods for this, with probably the most widespread technique in studies of ecology and evolution being the use of spectrometry. However, as discussed previously (Stevens *et al*. 2007; Stevens, Stoddard & Higham 2009), spectrometry has a number of major drawbacks, including being restricted to small point samples and hence being unsuitable for analysing the two‐ or three‐dimensional nature of many patterns, being difficult to use in the field, requiring close contact with the specimen and being strongly affected by changes in measurement angle and distance from the probe to the specimen (White *et al*. 2015).

Digital cameras offer an alternative to other approaches, being versatile tools for gathering data in a wide range of scenarios. Their substantial consumer base is also continually driving lower prices together with higher resolutions and dynamic ranges (the ratio between the lightest and darkest values the camera is sensitive to within one photo). This is in contrast to equipment such as spectrometers, which remain specialist in scope and beyond the financial means of many researchers with limited budgets. Digital cameras also overcome many of the limitations of other methods, being easy to use in both the laboratory and field, non‐invasive without requiring touching or being close to the specimen, and with vast potential to implement a range of computer science approaches to analyse entire colour patterns, visual scenes and two‐ and three‐dimensional properties of objects (Shapiro & Stockman 2001; Stevens & Cuthill 2006; Stoddard, Kilner & Town 2014; Allen & Higham 2015). Today, it is also relatively straightforward to use ultraviolet (UV)‐sensitive cameras, an important consideration given many animals perceive UV light.

Despite their advantages and substantial potential for scientific use, there are several considerations and steps required in order to use digital cameras and images properly (Stevens *et al*. 2007; Stevens, Stoddard & Higham 2009; Pike 2011). Briefly, photographs are optimised for human viewing on low‐dynamic range display media so that they have a nonlinear response to changes in light intensity or radiance, meaning that data from nonlinear images will almost always under‐ or overestimate true object values. In addition, images also need to be standardised to control for changes in lighting conditions. Furthermore, a linear response to radiance is a prerequisite to measuring a camera's spectral sensitivities (Lovell *et al*. 2005; Stevens *et al*. 2007; Pike 2011; Garcia *et al*. 2014), which is essential if one is to convert images to animal visual system spaces. These issues mean that digital images should not be used for making objective measures of brightness, colour or pattern, either within or between photographs without first undertaking appropriate calibration (Stevens *et al*. 2007). Yet studies of animal and plant visual signals still frequently fail to address these problems when using cameras, resulting in inaccurate or even flawed data. Although a variety of studies do use fully calibrated cameras for analysing a diversity of visual signals (e.g. Lovell *et al*. 2005; Spottiswoode & Stevens 2010; Allen, Stevens & Higham 2014; Stevens, Lown & Wood 2014a; Stoddard, Kilner & Town 2014), these remain relatively infrequent. Beyond this, many studies fail to utilise the full potential of digital image analysis, for example the possibility of transforming data into metrics corresponding to animal vision, or analysing an object's pattern.

Ultimately, a major reason for the issues outlined above is that software technology lags behind that of camera availability. Generating linearisation curves manually often requires expensive equipment, such as calibrated grey standards and/or a spectroradiometer, in addition to coding skills with expensive software suites such as matlab. However, Chakrabarti, Scharstein & Zickler (2009) found that a linear relationship between RAW images and radiance could be preserved in many camera models when extracted correctly with freely available DCRAW software (Coffin 2015). This eliminates an important stumbling block when making objective measures from digital images. However, until now, there has been no user‐friendly software program that enables researchers to normalise their images, incorporate multiple layers (for example visible and UV channels), convert to animal colour spaces and to measure images easily. Instead, researchers have needed to do much of this manually, including the sometimes complex calculations involved. Here, our aim is to address these issues with the release of a dedicated suite of tools, in freely available open source software. Below, we first give an overview of the toolbox (in addition to the associated user guide) and then demonstrate its accuracy for importing linear images, normalising them and converting to animal cone‐catch data.

## Image calibration and analysis toolbox overview

The following is a brief overview of the Image Calibration and Analysis Toolbox. Further detailed instructions and examples are included with the associated guide that can be downloaded freely along with the image processing toolbox at www.jolyon.co.uk or www.sensoryecology.com. The toolbox is a series of additions for the free open source imagej software (Schneider, Rasband & Eliceiri 2012), and the files need to be copied to the imagej ‘plugins’ folder. The toolbox is capable of both extracting linear images and performing image calibrations, as well as implementing a range of analytical tools for measurement.

### Equipment checklist

For making objective measurements of reflectance (brightness), colour or pattern in the human‐visible range all that is required is a consumer digital camera that can produce RAW images, a grey standard (or multiple standards), and a scale bar (required for pattern analysis across different photographs). For set‐ups that are restricted to human‐visible light (ca. 400–700 nm), a photography grey standard (there are various types available from most photography suppliers) is sufficient and readily affordable. For ultraviolet imaging, the standard must be grey into the UV spectrum (i.e. down to *ca*. 300 nm). For this, a sintered PTFE (polytetrafluoroethylene, e.g. Spectralon by Labsphere) grey standard is frequently used with reflectance values from around 5% to 99%. Artificial lighting is also more problematic for UV imaging as a single broad‐spectrum light source is required. Further information can be found in the dedicated user guide. Measurements from this set‐up using our toolbox are objective, but would be specific to the camera rather than a visual system (i.e. if one were to use a different camera, the results might be slightly different due to variation in the sensor sensitivity curves of different camera makes and models). Visual system‐specific results require a camera with known spectral sensitivities. These sensitivities are difficult to measure, so we have provided the sensitivities of a number of camera systems in the toolbox. Ultraviolet sensitivity is common in many taxa, such as birds, reptiles, amphibians, fish, stomatopods, insects and some mammals, meaning that a full‐spectrum‐converted camera is required to cover their sensitivities. In this instance, one photograph through a visible pass filter and a second through a UV pass filter can be combined by the software to cover the range of wavelengths required to model a non‐human visual system.

### Taking and processing photos

**Figure 1:**
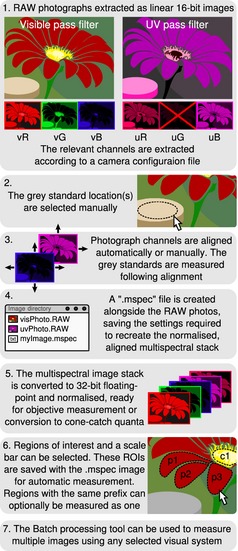
Multispectral image preparation example. This diagram outlines the important steps taken in order to create a normalised, aligned stack from a scene photographed in both visible and UV light. Quantitative measurements can be made from the resulting image, or it can be converted to animal cone‐catch quanta if the camera's sensitivity functions are known. The final step shows regions of interest being selected. The user can select whether the regions labelled ‘p’ for petal in this instance should all be measured individually, or whether they should be combined prior to automated measurement, enabling easy within and/or between region and photograph measurements. Channels are named with the lower case prefix denoting the filter type (e.g. ‘v’ for visible and ‘u’ for UV), and the suffix the camera's channel (e.g. R, G or B). Many more combinations can be created with additional filters.

Photographs must be taken in RAW format with one or more grey standards (either in the same photograph or a second photograph taken with identical lighting and camera settings: a ‘sequential method’; Stevens, Stoddard & Higham 2009). Once the photographs are transferred to a computer, the first step is to generate a ‘multispectral image’, being a stack of images taken at different ranges of wavelengths (‘channels’ hereafter). Standard colour photographs already comprise a stack of red, green and blue images, although this software can handle any number of additional channels. The script will guide the user through the various import options and requires specifying the camera and filter (if any) combination, the grey standard reflectance percentage value(s) and how to align the visible and UV images for UV photography. Standard filter combinations are included (e.g. ‘visible’ for standard red–green–blue (RGB) images and ‘visible & UV’ for UV images). Additional camera and filter combinations can be specified by creating a new configuration file, as detailed in the guide. Figure 1 shows the image preparation sequence for combing visible and ultraviolet (UV) photographs.

After specifying the import settings, prompts will ask the user to select the RAW image file(s), the relevant channels are then extracted from the RAW images at 16‐bits per channel, and the slices in the stack are aligned. Next, the script prompts the user to select the grey standard(s) by drawing a selection area (e.g. box, circle or custom polygon) over the grey standard(s). Multiple grey standards are recommended for bright field conditions to control for veiling glare (see below). The grey standard measurements and alignment offsets are saved in a .mspec file alongside the RAW images. This .mspec file saves all the information required to reload this multispectral image and is linked to the RAW files, so it must remain in the same directory as the RAW files.

The software then converts the multispectral stack to 32‐bits per channel and performs the normalisation, controlling for lighting changes between photographs and scaling pixel values to reflectance (this process takes around a second). When re‐opening an .mspec image, its associated RAW files are automatically imported, the channels are arranged and converted to a 32‐bit stack, and the pixels are normalised and aligned. This process takes just a few seconds depending on the number of channels, resolution and computer. At this point, reflectance values and colours in the normalised image can be measured objectively and regions of interest (ROIs) can be drawn over the parts of the image to be measured. Pattern analysis requires uniformly scaled images if patterns are being compared between different photographs (i.e. the images must have the same number of pixels per unit length). To use the automated scaling, the user can use the line tool to draw a line along a known scale bar in the image, press the ‘S’ key and specify the length of the ruler when prompted. The toolbox resizes the image to a scale set by the user automatically before pattern measurement. Bird egg colours and patterns are commonly measured in brood parasitism (e.g. Spottiswoode & Stevens 2010; Stoddard & Stevens 2010), camouflage and evolutionary studies (Kilner 2006). To facilitate egg measurement, the toolbox also includes an egg measurement and selection tool that allows the user to click a few points around the edge of an egg (or a similarly shaped object) and to model the curvature of the egg and calculate its shape and volume metrics (Troscianko 2014). The scale bar and regions of interest are saved alongside the .mspec file and are automatically reloaded when the .mspec image is opened. Batch image processing tools included in the toolbox allow automated measurement of the colour, luminance and pattern properties from multiple image selections in a folder with any number of .mspec images (see Fig. 1). Once the colours, patterns and luminance distributions of regions have been measured, there are further tools for conducting analyses, for example calculating pairwise difference values between colours from cone‐catch images using models of visual discrimination (Vorobyev & Osorio 1998; Siddiqi *et al*. 2004), or pattern difference analyses.

## Toolbox technical descriptions and testing

### Extracting linear images

RAW photographs are proprietary file formats specific to each camera manufacturer that preserve the analog‐to‐digital photosensor responses in a 12‐ or 14‐bit format, and each manufacturer uses their own file extension (e.g. ‘.CR2’ or ‘.NEF’ for Canon and Nikon, respectively). Red, green and blue photosensors are arranged in a non‐overlapping mosaic on the camera sensor, so these must be demosaiced such that each pixel has all three colour values. dcraw (Coffin 2015) is an open source software package that can read the RAW files from most camera manufacturers, perform the demosaic and, most importantly, extract the pixel values in a linear fashion (Chakrabarti, Scharstein & Zickler 2009) and output a 16‐bit image. This negates the need to manually calculate the nonlinearity of a given camera and calculate a linearisation equation (as specified in Stevens *et al*. 2007). Our toolbox utilises ij‐dcraw (Sacha 2013), a plugin for imagej that uses dcraw (Coffin 2015) to extract pixel data from RAW files linearly.

### Testing linearity methods

Eight off‐the‐shelf consumer digital cameras were used to test the linearity of extracted images. The cameras consisted of four Nikon D7000s having undergone full‐spectrum quartz conversion (see below, two with Nikon Nikkor 105 mm lenses, one with a Coastal Optics 60 mm quartz lens and one with a Coastal Optics 105 mm quartz lens), a Nikon D90 (with Nikon Nikkor 105 mm lens, having undergone quartz full‐spectrum conversion), a Canon 5DMKII (not converted), a Canon 7D (with quartz full‐spectrum conversion, both with a Canon 50 mm f/1·4 lens) and a mirror‐less Samsung NX1000 (converted to full spectrum with no quartz filter, fitted with a Pentax Asahi Super Takumar 50 mm). A set of eight diffuse Spectralon reflectance standards (with percentage reflectance values of 2, 5, 10, 20, 40, 60, 80 and 99) was photographed in a dark room, illuminated from a distance of 2 m at right angles to their surfaces with an Iwasaki eyeColour arc lamp that has a broad emission spectrum designed to simulate the CIE (International Commission on Illumination) recommended D65 illumination. The bulb was modified to remove its UV filter (using a steel brush drill bit to remove the coating from the bulb), enabling UV photography while replicating natural illumination conditions. Standard reflectance values were verified using a Jeti Specbos 1211 UV spectroradiometer, measuring reflectance relative to the 99% standard from 300 to 700 nm. All photographs were taken in RAW format using an ISO of 400. Exposures were selected that had 99% standard pixel values as close to the maximum (saturation) level as possible, and photographs were taken across the whole range of available aperture stops (7 or 8 photographs depending on the lens model), resulting in a number of photos from each camera taken across a wide range of integration times (‘shutter speeds’ hereafter). In order to test for the linearity of pixel responses across a large dynamic range, we modelled pixel values for each channel against calibrated standard reflectance values. Reflectance values were first multiplied by the pooled average grey standard pixel response for that photograph to control for light intensity differences between different apertures. This allowed us to determine whether linearity holds within and between images of different shutter speeds.

### Linearity results

**Figure 2:**
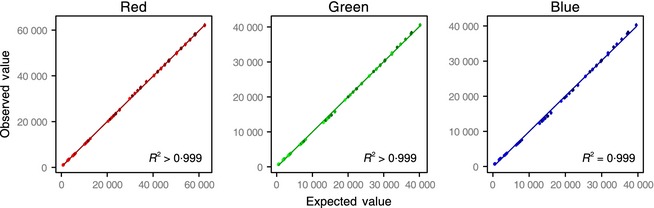
Example of camera linear responses from a Nikon D7000. Expected reflectance values normalised between exposures (*x*‐axis) are plotted against mean observed pixel values measured for each standard (*y*‐axis). This normalisation allows us to test for within and between photograph linearity across a wide range of apertures and shutter speeds while preserving the actual pixel value on the *y*‐axis, allowing us to detect any hardware‐based nonlinearity at specific pixel values. Error bars show mean ±1 standard deviation; point brightness is scaled with aperture.

Linear regressions between pixel values and normalised reflectance values were found to be near perfect fits across all eight reflectance standards and shutter speeds (see Fig. 2). Coefficients of determination (*R* ^2^ values) were all >0·998 for all camera models and colour channels (mean = 0·999, median >0·999). These findings improve on the precision of previous authors who found images extracted linearly with dcraw are indeed linear (Chakrabarti, Scharstein & Zickler 2009; Akkaynak *et al*. 2014). All images will contain some level of noise (which are dependent on the sensor, its ISO gain, and light levels reaching the sensor causing shot noise). RAW images do not support negative numbers, so noise will artificially increase the pixel values near zero, limiting the camera's dynamic range. While our lowest reflectance standard (2%) maintained linearity, measurements nearer zero will become less reliable and artificially increased as the sensor's signal to noise ratio decreases. This does not mean the software cannot be used to measure dark objects, but does imply that very dark and light objects cannot be measured in the same photograph (the dynamic range of the camera becomes limiting). When measuring dark objects, grey standard(s) with a low level of reflectance should be used. Photographing the same scene with a shorter exposure would then allow measurement of lighter objects, similar to the principles of high‐dynamic range photography. However, in practice, most biological objects do not have such extremely low reflectance values.

## Normalisation

Natural and artificial light sources (illuminants) vary in intensity and colour (Endler 1993), meaning two photographs taken at different times, or in different habitats, can rarely be compared without controlling for these lighting changes (even under laboratory conditions). Normalisation of digital images controls for differences in illumination between photographs by scaling each of the colour channels to a uniform reflectance level. Grey or white standards of a known reflectance are used in the image to measure the responses of each channel and correct them accordingly in a manner similar to a visual system's chromatic adaptation to ambient lighting (Stevens *et al*. 2007). This is similar to setting the ‘white balance’ in photography; however, this operation requires linear images and so is not performed correctly in photography software packages that offer a white balance eye dropper tool on nonlinear images designed for viewing on prints and screens. The ideal standard should have a flat reflectance spectrum across the wavelengths being photographed (i.e. grey) and should be highly diffuse (i.e. demonstrate Lambertian reflectance, scattering reflected light equally in all directions). Grey standards around the 20–50% level have the advantage that they are less likely to be inadvertently over‐exposed (Stevens *et al*. 2007), and are used routinely in photography. However, white standards (e.g. 95–99% reflectance) are advantageous because they can more easily be used to set the image's exposure with on‐screen histograms (judging the exposure from the highest peak in the histograms). The angle of the grey standard(s) relative to the illuminant and camera are extremely important, particularly when the illuminant is a point source (highly directional) rather than diffuse. When the object being measured is roughly planar (such as a butterfly wing), the grey standard should be in the same plane as the object and at the same angle. If the object being measured is more complex then the angle of the standard should be based on the direction of the illuminant, either directly facing the illuminant if it is an artificial point source, or level with the ground under natural daylight conditions (assuming the surfaces being measured are more likely to be horizontal in this case). Whatever grey standard angle rules are decided upon, it is critical that this is kept uniform across photographs and treatments. Grey standards in linear images can also be used to measure colour or intensity changes in the illuminant itself. Illuminant colour can be measured from the relative ratios of the linear RGB values, or, if exposure time is controlled for the intensities between photographs can also be measured (see Lovell *et al*. 2005 and Arenas, Troscianko & Stevens 2014).

The toolbox calculates normalised pixel values *V*_norm_ from their linear pixel values *V*_linear_, the grey standard reflectance value *S*, the maximum bit range value (in this case the unsigned 16‐bit maximum) and the mean pixel values for the grey standard *G*:

(eqn 1) $$V_{\mathrm{norm}}=V_{\mathrm{linear}}\frac{\left(\frac{S}{100}\right)\;65,535}{G}$$

### Multiple grey standards

No lens can completely eliminate internal reflections and light ‘bleeding’ onto the sensor from undesired sources, resulting in optical veiling glare (McCann & Rizzi 2007). This effect is most prominent in ‘lens flare’ artefacts produced when photographing into or near a light source such as the sun, but can be impossible to detect when the artefacts are less extreme. Optical veiling is more likely to manifest in bright field conditions than a laboratory dark room, and even more common when using UV filters, resulting in an increased black point and reducing the camera's dynamic range (and the photograph's contrast). Calculating a photograph's black point requires two or more grey standards – ideally with reflectance values near the top and bottom of the camera's range (i.e. a black and a white standard). We have therefore added the ability for the toolbox's normalisation protocol to utilise any number of grey standards so that the black point can be estimated. The software prompts the user to input the reflectance values of the standards used and then asks them to draw a selection area over each respective standard. A linear regression is then plotted between the mean measured standard values, and the normalised pixel values are calculated from the resulting straight line equation (i.e. eqn 1, plus an intercept). If more than two standards are used the script warns the user if the linearisation fit is lower than 0·98. In circumstances where multiple standards are not available and veiling glare could be a concern the toolbox can also estimate the dark point based on the channel's histogram by assuming that the lowest 0·5% of pixels have a reflectance of 0·5% (the absolute lowest pixel value is not used because it will be highly susceptible to noise).

### The sequential method

Sometimes, the reflectance standard cannot be placed directly into the image being measured, for example, when photographing a free‐ranging animal before it moves away. In the light of this, the toolbox offers an option for selecting a grey standard in a separate photograph taken under identical conditions as the target (Stevens, Stoddard & Higham 2009). When using this option, it is essential that there are no lighting changes or camera setting changes between photographs, so the photographs should be taken as fast as practically possible, and avoiding changeable lighting conditions such as patchy cloud. Further details are available in the guide.

## Mapping to cone‐catch values

Many studies analysing visual signals seek to calculate predicted photoreceptor responses (cone‐catch values) of animal visual systems and then to utilise this in further models. The spectral sensitivities of a camera's red, green and blue channels vary between models and will not match human long‐wave, medium‐wave and short‐wave (LMS) receptor sensitivities perfectly. The colours generated by a camera and lens combination are therefore device dependent, and a mapping function must be used to convert from camera colours to standardised colorimetric values or visual system specific data (such as LMS or CIE XYZ space, or cone‐catch values) (Hong, Luo & Rhodes 2000; Westland, Ripamonti & Cheung 2004; Lovell *et al*. 2005; Stevens & Cuthill 2006; Stevens *et al*. 2007; Pike 2011). In theory, these functions are estimates subject to error, and cannot rule out metamerism, where, for example, two different colours viewed under the same illumination could produce the same RBG values, but could produce different LMS values. Metamerism is exacerbated by spectra that have particularly sharp steps in reflectance across different wavelengths. However, natural spectra tend to be smooth enough for accurate polynomial mapping with very low degrees of error (Hong, Luo & Rhodes 2000; Lovell *et al*. 2005; Stevens *et al*. 2007; see below). Our toolbox generates these mapping functions by estimating camera RGB and visual system cone‐catch values (e.g. human LMS values or CIE XYZ space; see below for mapping to non‐human photoreceptors) from a library of natural spectra under a specific illuminant. The following examples show the calculation of long‐wave cone‐catch values (quanta, *L*_c_), and these are repeated for all camera and receptor channels:

(eqn 2) $$L_{c}=\sum_{\lambda \max}^{\lambda \min}l_{\lambda }Q_{\lambda }I_{\lambda }$$

where *l*_λ_ is the long‐wave sensitivity, *Q*_λ_ is the spectral reflectance of the sample, and *I*_λ_ is the illuminant's spectral radiance at wavelength λ. Cone‐catch quanta are then normalised using eqn 3 to *L*_n_ so that a grey surface viewed under illuminant *I* has equal cone‐catch quanta in all channels according to the von Kries coefficient law of chromatic adaptation (i.e. incorporating an aspect of colour constancy, which is also standard in methods based on reflectance spectrometry), where *L*_wr_ is the cone‐catch quanta calculated for a white reference:

(eqn 3) $$L_{n}=\frac{L_{c}}{L_{\mathrm{wr}}}$$

A multiple regression approach is then used to create the mapping functions for each receptor channel from the camera's responses. The number of interactions and transformations in the polynomial can vary (see Hong, Luo & Rhodes 2000), for example:

(eqn 4) $$L=a_{1}R+a_{2}G+a_{3}B+a_{4}\text{RG}+a_{5}\text{RB}+a_{6}\text{GB}$$

(eqn 5) $$L=a_{1}R+a_{2}G+a_{3}B+a_{4}\text{RG}+a_{5}\text{RB}+a_{6}\text{GB}+a_{7}\text{RGB}+a_{8}R^{2}+a_{9}G^{2}+a_{10}B^{2}$$

In this example, there are three camera colour channels and three cone types. However, additional channels can be added to these polynomial functions if more camera channels are available. The addition of more camera channels and higher level interactions can make these models large and slow to converge and subsequently slow to apply to an image. Our cone mapping script therefore offers tools to set the maximum number of interactions (two and three way, respectively, in the examples above), whether to include square transforms, and can also use stepwise model simplification to remove terms from the model that do not improve the model's fit given the degrees of freedom they consume, thus reducing model overfitting. This model simplification can be based optionally on the Akaike Information Criterion (AIC) or Bayesian Information Criterion (BIC), with the latter being more conservative, removing more model terms. One advantage of polynomial colour mapping is that once the model is generated – which takes a few minutes and needs to be calculated only once for each camera and visual system combination – processing each multispectral image to cone‐catch quanta images is extremely fast (15–20 megapixels per second for 32‐bit blue tit cone‐catch quanta on an Intel i5 laptop). Cone mapping models can be generated by preparing a camera's sensitivity functions in a .csv file according to the guide and then running ‘Generate Cone Mapping Model’. This script will ask the user to specify the camera, the illuminant, the receptor sensitivities of the desired visual system, the library of natural spectra, the model interaction level and simplification protocol (if any). The script then generates the camera and receptor cone‐catch quanta and automatically uses R (R Core Team 2013) to process the data in a linear model for each receptor. The script then retrieves the models from R and compiles them into a script that integrates with the other functions in the toolbox. *R* ^2^ values are also extracted to judge the quality of fit in each receptor.

The number of camera colour channels should be equal to or greater than the number of receptors being mapped to, and their sensitivities should cover the same spectral range. For example, a camera with three channels (RGB) can reliably map to trichromatic humans, dichromatic mammals or achromatic dogfish cone catch, but it would not be possible to map to species that see into ultraviolet (UV), or have particularly narrow spectral sensitivities. The model *R* ^2^ values can be used to determine the quality of the mapping model.

**Figure 3:**
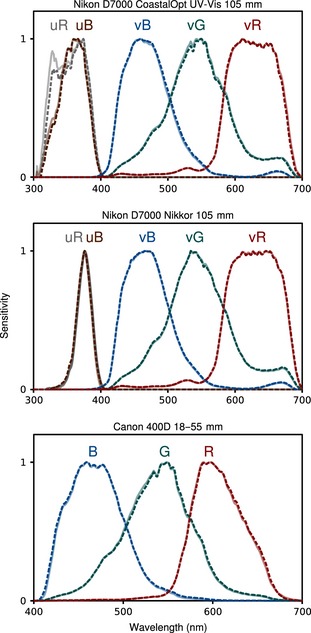
Spectral sensitivities of duplicate camera set‐ups. Solid lines and dashed lines show the spectral sensitivities of two different set‐ups that use the same equipment and – in the case of the D7000s – have undergone the same full‐spectrum conversion. The sensitivities are almost identical between all set‐ups, suggesting that these spectral sensitivity functions could be used for other identical set‐ups.

Many animals can see wavelengths below the ~400 nm limit of humans, meaning ultraviolet (UV) photography in addition to human‐visible photography is required for modelling their vision. Most camera sensors are sensitive to UV wavelengths to some degree once their UV‐blocking filters are removed (and replaced with a UV transparent filter if required). Our toolbox contains the spectral sensitivities of a number of camera, lens and filter combinations with and without this conversion for researchers who obtain the same equipment (see Fig. 3 for between set‐up comparisons). Companies exist that can undertake the full‐spectrum conversion process (e.g. Advanced Camera Services, Thetford, UK). Alternatively, the filter can be removed without needing to be replaced with a quartz sheet as long as the sensor can be moved sufficiently to restore focusing to infinity (e.g. see http://www.jolyon.co.uk/2014/07/full-spectrum-nx1000/). A full‐spectrum converted camera will be sensitive to a large band of wavelengths from UV to near infrared, although the sensitivity to infrared is generally far higher than UV, and extends further (often up to around 900 nm). Filters are therefore required to photograph the relevant wavelengths at shutter speeds that reflect the camera's sensitivity to those wavelengths (e.g. UV sensitivity is often around 100 times lower than human‐visible light). On‐screen histograms in camera live‐view modes are useful for judging the correct exposure times. For UV photography, we use Baader Venus‐U filters, transmitting wavelengths between ~320 and 380 nm, and to take photographs in the human vision range with the same cameras, we use Baader UV/IR cut filters, transmitting wavelengths between ~400 and 680 nm. Both filters can be purchased over the internet and come in 2 inch sizes that, when combined with appropriate filter holders, can be attached to most lenses. We built custom plastic filter slides to enable us to switch quickly between filters with minimum disturbance to the camera's focus and position (CNC G‐Code machining scripts for the sliders can be made available on request). UV photography also requires a lens that transmits ultraviolet wavelengths, and most standard lenses do not do this. The standard Nikon Nikkor 105 mm lens transmits UV wavelengths down to approximately 360 nm; however, it is not achromatic between visible and UV wavelengths (an ‘achromatic’ lens is designed to limit radial chromatic distortion between given wavelength ranges, so a visible–UV achromatic lens will not require refocusing for sharp visible and UV photographs). Coastal Optics 60 mm and 105 mm lenses (which are both achromatic across the UV and visible range, available from Jenoptik) have transmission below 300 nm. These latter lenses therefore cover a greater portion of the UV spectrum, but are considerably more expensive. A few out‐of‐production lenses were made with UV transmitting glass and can be purchased second‐hand online, see Verhoeven & Schmitt (2010). These include the Novoflex Noflexar 35 mm and Nikkor EL 80 mm (metal body version only). Both have transmission down to approximately 320 nm and are good achromats. However, sourcing a pristine version would be essential. Visible‐band and UV‐band photographs can then be combined into a multispectral image stack to enable mapping of UV‐sensitive visual systems.

### Alignment

Each channel that makes up a multispectral image stack needs to be perfectly aligned with the others for accurate colour measurements, particularly when measuring the colours of small objects subtending a few pixels. When changing filters in field conditions the camera is often knocked slightly, throwing out the alignment. In addition, non‐achromatic lenses need refocussing in different wavelength bands. This refocussing has the undesirable effect of zooming the image slightly, making alignment more problematic. The toolbox offers various alignment options to choose from when preparing a multispectral image. When aligning any two photographs, the nearest channels (in terms of mean spectral sensitivity) should be used for alignment. These channels are specified in a camera configuration file that also contains the order in which channels should be extracted. Manual alignment prompts the user to use their mouse to drag one image over the other until optimal alignment is reached.

An automated alignment process was also developed that can find the correct scaling if the lens required refocussing. Existing image alignment tools developed for image stitching were evaluated, but found to generate unreliable alignments between the UV‐blue and visible‐blue channels (the closest channels in terms of spectral sensitivity). Thus, an exhaustive alignment and scaling algorithm was written that can find the optimal planar alignment and scaling factor between photographs. The script first aligns the two selected colour channels by calculating the lowest sum of absolute pixel differences across the whole image in a three‐by‐three matrix at a given offset (e.g. 128 pixels), it then centres on the best alignment and halves the pixel offset (e.g. to 64 pixels). This continues until the offset reaches one. The script then scales one of the images (e.g. by 1%) and re‐aligns as above. If the quality of the alignment is superior at the new scale, it carries on in this direction, halving the scale value (e.g. to 0·5%); otherwise, it scales in the opposite direction and so on until it finds the best scale and alignment. This algorithm is fairly slow (approximately one minute on a modern laptop depending on the chosen settings), but reliably produces alignments far superior to those achieved manually, even with considerable movement of objects in the scene (e.g. foliage swaying in the wind). This automated alignment requires images with sufficient detail to inform the alignment. Large plain surfaces, for example, have few details for the alignment to fit. Therefore, an additional option allows the user to specify a region to align that could be the main area of interest, or contain salient image features. Following alignment, the program flips between the aligned slices for a visual inspection of the alignment quality.

### Testing mapping methods

In order to determine the accuracy of camera‐based cone‐catch quanta, we performed tests on 12 different camera, lens and filter combinations for a range of visual systems across birds, mammals, fish and insects. The visual systems included tetrachromats: blue tit *Cyanistes caeruleus* (Hart *et al*. 2000), peafowl *Pavo cristatus* (Hart 2002); trichromats: human CIE XYZ and honeybee worker *Apis mellifera* (Peitsch *et al*. 1992); dichromats: ferret *Mustela putorious furo* (Calderone & Jacobs 2003; Douglas & Jeffery 2014) and pollack *Pollachius pollacbius* (Shand *et al*. 1988); and an achromatic dogfish *Scyliorhinus canicula* (Gačić *et al*. 2007). The spectral sensitivities are provided for these species in the toolbox but users should cite the relevant sources above for the data used in the mapping. Sensitivities have been linearly interpolated to 1 nm increments where these data were not available. The quality of fit was judged both from the mapping models reported fit to the natural spectrum data base and to a number of colour samples measured with both camera and spectroradiometer.

The cameras, lenses and filters used are listed in Table S1 (Supporting information) and see Fig. 3. We use a channel naming convention whereby the lower case prefix describes the filter used (e.g. ‘v’ for visible, ‘u’ for UV), and the suffix refers to the camera's sensor channel (i.e. R, G or B). Spectral sensitivities were determined using a method similar to Lovell *et al*. (2005) and Garcia *et al*. (2014). However, instead of taking multiple photographs of monochromated light at different wavelengths, we split a collimated beam (i.e. parallel rays of light) of broadband white light from an eyeColor arc lamp (modified to remove it's UV‐blocking coating as above) through a pair of fused silica prisms, projecting the resulting ‘rainbow’ onto a sintered PTFE sheet. A single photograph of the resulting spectrum can be used together with known spectral irradiance (measured with a Jeti Specbos 1211 UV spectroradiometer) to calculate spectral sensitivities. Full methods for this approach are beyond the scope of this article and will be published separately; however, the results are in accordance with other methods and result in good fits (see below, Table S1 and Fig. 3). Spectral sensitivities can be inferred using other methods (Pike 2011); however, this requires a large number of diffuse samples with known and subtly varied reflectance profiles (which is particularly problematic for UV) and makes the assumption that sensitivities can be described by curve functions, which is not always the case (see Fig. 3).

**Figure 4:**
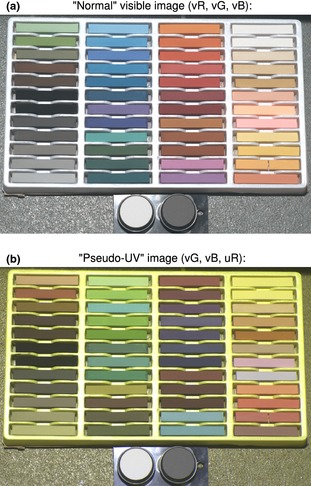
Colour sample measurements. This set of 48 colour pastels was used to compare camera and spectroradiometer measurements of cone‐catch quanta. Image ‘a’ is a normal human‐visible photograph, and image ‘b’ is a false colour combination of green, blue and UV channels (vG, vB and uR). Both images were square‐root‐transformed from 32‐bit linear, normalised images to display correctly on low‐dynamic range media. Standard colour charts have poor UV reflectance presumably to reduce colour fading with age, while these pastels have varied reflectance spectra in UV. For example, the pastels that appear blue in image ‘b’ have UV peaks relative to green and blue.

Cone mapping models were generated using a library of 3139 natural spectra. Of these, 2361 reflectance spectra were from the Floral Reflectance Data base (Arnold *et al*. 2010), and the remainder were collected from bird eggs, bird plumage, insects, minerals, tree bark and vegetation (unpublished data). As stated above, this library is included with the toolbox, but users should cite Arnold *et al*. (2010) and this article as the source of these spectra. All spectra ranged from 300 to 700 nm (blue tit, peafowl, honeybee and ferret) or 400–700 nm (human XYZ, pollack and dogfish) at 1 nm increments, and a D65 illuminant was used. Trichromatic, dichromatic and achromatic models were fitted with three‐way interactions, with no model simplification and no square transforms. Tetrachromatic models were fitted similarly, but with a maximum of two‐way interactions, see Table S1. A set of 48 colour pastels (Royal Langnickel) was used as a colour chart for comparing cone‐catch quanta with the camera and spectroradiometer. Standard photography colour charts have poor UV reflectance properties – presumably to prevent UV from fading the colours over time. These pastels cover a wide range of colours and display interesting UV reflectance peaks, as highlighted in Fig. 4. Comparing cone‐catch quanta for these colours is likely to be a conservative test of colour reproduction given the selection of complex, saturated colours when compared to most natural spectra. Pastel reflectance spectra were measured as above with a spectroradiometer and were photographed under an Iwasaki eyeColor arc lamp designed to resemble D65 emission spectra. 20% and 80% reflectance Spectralon standards were used to normalise the images (see above).

### Mapping results

**Figure 5:**
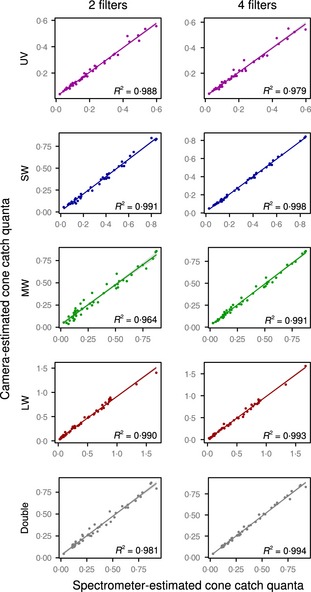
Blue tit cone‐catch quanta estimates for a range of pastel colours, comparing spectrometer and camera estimates. Cone‐catch estimates from a Canon 7D with two filters and five channels (i.e. vR, vG, vB, uB and uR) are shown in the left column, and estimates from the same camera with four filters and seven channels are shown in the right column (i.e. rR, gR, gG, bG, bB, uB and uR). Shaded areas show standard error. Cone‐catch estimates with two filters are exceptionally good; however, the additional channels provide some greater spectral partitioning in the medium‐wave range, improving the quality of fit most for this channel. Two of the pastel colours were fluorescent – absorbing short wavelengths and emitting them in the long‐wave range. These two points highlight the ability of the toolbox to work with values outside of the 0–100% reflectance range.

The mean *R* ^2^ values for fits between camera cone‐catch quanta and receptor cone‐catch quanta for the data base of natural spectra across all camera configurations and visual systems was 0·999 (median >0·999), with a minimum of 0·996. These results suggest polynomial cone mapping functions can reliably convert camera colour measurements to cone‐catch quanta from natural reflectance spectra. Comparisons between cone‐catch estimates of complex pastel colours from camera and spectroradiometer also suggest a good fit; the mean fit across all cameras and visual systems was 0·981 (median = 0·983), with a minimum of 0·951 (see Table S1 and Fig. 5), showing that the mapping approach is highly accurate compared to more widespread spectrometry‐based approaches.

## Image analysis

We recommend the use of the toolbox's ‘Batch Multispectral Image Analysis’ tool for most image measurements. This ensures related images are measured with exactly the same settings, and the computer can be left alone when processing large data sets. The user is asked to specify the directory containing the .mspec images (with their linked RAW files in the same directory). Various options are then presented for mapping to a given visual system (or no visual system). If regions of interest (e.g. squares, polygons and so on; see Fig. 1) have been added to the .mspec image, these are measured individually, or pooled together if specified (overlapping regions are also unified in this case). If no regions have been specified with the .mspec file, the whole image is measured. Scale bars added to the image can be used to automatically scale all images in the batch analysis to a uniform number of pixels per unit length, which is required for most pattern analyses. We provide tools for performing some pattern analyses based on Fast Fourier bandpass filtering. This form of analysis is increasingly widely used to measure animal markings (e.g. Godfrey, Lythgoe & Rumball 1987; Stoddard & Stevens 2010) and is loosely based on our understanding of low‐level neurophysiological spatial scene processing in numerous vertebrates and invertebrates. Briefly, images are filtered to set of spatial frequencies, and then, the ‘energy’ at each frequency band is measured as the standard deviation of the filtered image (a granularity analysis: Chiao *et al*. 2009; Stoddard & Stevens 2010; see the guide for further information). The toolbox can provide basic descriptive pattern statistics (corresponding to attributes such as marking size, contrast and diversity; e.g. Stoddard & Stevens 2010) or can perform pairwise pattern difference calculations between two objects or samples. We also provide tools to perform colour analysis, including the widely used receptor noise‐based model of visual discrimination that can calculate the ‘just noticeable difference’ (JND) values between samples (Vorobyev & Osorio 1998; Siddiqi *et al*. 2004). This can be used to determine whether two samples are likely to be discriminable to a given visual system. These colour and pattern analysis tools are described in detail in the dedicated user guide.

## Discussion

The field of visual ecology is rapidly growing (Cronin *et al*. 2014), and the availability of digital imaging makes the testing of numerous aspects of animal and plant coloration, signalling and camouflage available to almost any researcher. In addition, image analysis is widely used in medical studies, forensics and beyond. However, digital photographs are nonlinear with respect to radiance and require a range of calibrations, meaning one cannot make quantitative measurements from pixel values without first transforming those images. Some authors and reviewers remain unaware of these issues and continue to use software such as Adobe Photoshop to measure pixel values and test RGB values from unstandardised images, not to mention frequently failing to account for the visual system of their target species. To date, the linearisation process has generally required expensive, specialist equipment and coding skills (Barnard & Funt 2002; Stevens *et al*. 2007; Garcia *et al*. 2013). Our image processing toolbox overcomes these problems by extracting linear images from RAW files (Chakrabarti, Scharstein & Zickler 2009; Akkaynak *et al*. 2014; Coffin 2015).

The toolbox provides functions that control for lighting conditions in a more flexible way than previous methods (Stevens *et al*. 2007), by allowing two or more grey standards to be used for estimating reflectance in addition to the photograph's black point. This controls for low‐level optical veiling glare (McCann & Rizzi 2007) and also enables measurements of reflectance in photographs taken through slightly opaque media (such as fog, haze or murky water), or through transparent surfaces (such as still water or glass) on the condition that the details being reflected are suitably uniform (such as a clear or uniformly overcast sky), and the grey standards are next to the objects being measured.

Previous image analysis studies have also almost exclusively used 8‐bit images, which can impose serious limits on the effective dynamic range and precision available, particularly in linear images because natural scenes tend to have a log distribution of reflectance levels. In practice, this means that the vast majority of pixels in a linear 8‐bit image cluster below pixel levels of about 30–40 of 255. The normalisation process of previous methods was also unable to accommodate reflectance values >100% relative to the grey standard. However, such levels are common in any natural scenes that have shiny (non‐Lambertian) surfaces, for example leaves and wet pebbles, as well as in cases of fluorescence or when objects emit or transmit light through them (for example, a leaf viewed against the sky). We have overcome this problem by importing the images in 16‐bit (preserving the camera's entire dynamic range) and then performing the normalisation process and subsequent processing on 32‐bit floating point images. This allows reflectance values >100%, removing the possibility of data loss and saturation in image post‐processing. This also enables the measurement of shiny, luminous or fluorescent surfaces, and any situation where the grey standard(s) receives a lower level of illumination than other parts of the scene (as will be common in dappled shade for example).

Saving 32‐bit images would take up an unwieldy amount of space on hard drives because the multispectral stacks are often many hundreds of megabytes. However, the speed with which normalised or cone‐catch multispectral images can be reconstructed from their RAW files means that instead of saving these huge files, a small ‘.mspec’ file is used that saves all of the required information. Loading the images straight from RAW also makes it difficult to inadvertently apply incorrect or repeated image transformations because the RAW files cannot be edited. RAW files are also the most suitable format for backup and archiving purposes because it is clear that no potentially lossy post‐processing has been applied, and the photograph's meta‐data are preserved.

Many visual ecologists work on hypotheses that relate to non‐human visual systems. Our toolbox provides functions for mapping from camera colour to cone‐catch quanta of almost any visual system (we supply a range of model visual systems and hope to build up a larger data base). The two main requirements for cone mapping are that the photographs cover the spectral sensitivity range of the target visual system (for example, visible and UV bands for birds, reptiles, many insects and so on) and that the spectral sensitivities of the camera are known (Lovell *et al*. 2005; Stevens *et al*. 2007; Pike 2011; Garcia *et al*. 2014). Determining these spectral sensitivities still requires specialist equipment, and further work should make this process more accessible to researchers, or manufacturers should be encouraged to publish this information. We plan to publish additional methods on this approach in time. In addition, we supply the software with spectral sensitivity curves for the camera, lens and filter combinations that we have characterised for spectral sensitivity so far. Our current work suggests, at least for the Nikon D7000s and Canon D400s, that spectral sensitivity curves do not vary greatly from one camera to the next provided it is of the same make and model, fitted with the same lens and filters and (if applicable) has undergone the same full‐spectrum conversion (see Fig. 3). Thus, researchers are free to use the spectral sensitivity curves we provide if they use identical set‐ups. However, until more camera set‐ups are characterised, this should be done with a degree of caution. Nonetheless, with these requirements met, the toolbox can combine multiple photographs into an aligned multispectral stack and then transform this to animal cone‐catch quanta. Previous research has demonstrated that camera RGB measurements can successfully be converted to human LMS cone‐catch quanta with polynomial mapping functions (Lovell *et al*. 2005; Stevens *et al*. 2007) and to the cone‐catch values of non‐human animals (Stevens & Cuthill 2006; Pike 2011; Stevens, Lown & Wood 2014b). We demonstrate that these models can reliably reconstruct cone‐catch quanta from a much larger number of camera channels, but that increasing the spectral resolution with more filters and channels is likely to provide diminishing returns in terms of model accuracy (see Fig. 5). The quality of these models also suggest hyperspectral imaging (Chiao *et al*. 2011), which is currently extremely expensive, and has low resolution and/or slow capture times, currently offers minimal benefits in terms of cone‐catch quanta measurements compared to consumer cameras fitted with just two filters.

Finally, the toolbox offers integrated measurement tools that can measure the colours, luminance and patterns of selected regions and then model the differences between chosen samples. By releasing these user‐friendly tools in one comprehensive package using open source code and software platforms (Schneider, Rasband & Eliceiri 2012; R Core Team 2013), we hope to encourage more researches to use digital cameras for making objective measurements and to exploit the vast potential that image analysis has to address a wide range of questions in biology.

## Supporting information


**Table S1.** Cone mapping model *R* ^2^ fits for numerous camera, lens, and filter combinations for a range of visual systems from tetrachromatic birds to achromatic dogfish.
