# Supplementary material for: Image calibration and analysis toolbox – a free software suite for objectively measuring reflectance, colour and pattern
Source: Methods Ecol Evol. 2015 Aug 6;6(11):1320–31. doi: 10.1111/2041-210X.12439 (PMC4791150; doi:10.1111/2041-210X.12439)
Supplement: Supplementary file 1 — Table S1. Cone mapping model R 2 fits for numerous camera, lens, and filter combinations for a range of visual systems from tetrachromatic birds to achromatic dogfish. [file MEE3-6-1320-s001.docx]

SUPPORTING INFORMATION

*Table S1*; Cone mapping model R^2^ fits for numerous camera, lens, and filter combinations for a range of visual systems from tetrachromatic birds to achromatic dogfish. The models report very good fits between camera and cone catch quanta for a library of natural spectra. The agreement between camera and spectroradiometer measurements of cone catch quanta for a chart of 48 colours (see figure 4) are also a good, particularly given the complex reflectance spectra of these colours. Filters are coded by letters; “v” is the Baader UV/IR cut filter, “u” is the Baader Venus-U filter, and “r,g,b” is a set of Baader narrowband CCD filters. All cameras listed have undergone a quartz full-spectrum conversion with the exception of the Samsung NX1000 (which was converted to full spectrum without a quartz sheet), and the Canon 400Ds, which were unmodified. Note that the poorest fits are in the MW channels, presumably because the green channel sensitivity in the cameras are more broad than animal MW sensitivities. The use of narrowband RGB filters improves the camera's ability to estimate MW cone catch quanta.
